# 10-Year Cardiovascular Disease Risk Estimation Based on Lipid Profile-Based and BMI-Based Framingham Risk Scores across Multiple Sociodemographic Characteristics: The Malaysian Cohort Project

**DOI:** 10.1155/2018/2979206

**Published:** 2018-07-17

**Authors:** Boekhtiar Borhanuddin, Azmawati Mohd Nawi, Shamsul Azhar Shah, Noraidatulakma Abdullah, Syed Zulkifli Syed Zakaria, Mohd Arman Kamaruddin, Chandralekah S. Velu, Norliza Ismail, Mohd Shaharom Abdullah, Salywana Ahmad Kamat, Afifah Awang, Mariatul Akma Hamid, Rahman Jamal

**Affiliations:** ^1^UKM Medical Molecular Biology Institute (UMBI), Malaysia; ^2^Department of Community Health, Faculty of Medicine, Universiti Kebangsaan Malaysia, Malaysia; ^3^Department of Paediatrics, Faculty of Medicine, Universiti Kebangsaan Malaysia, Jalan Yaacob Latif, Cheras, 56000 Kuala Lumpur, Malaysia

## Abstract

Cardiovascular disease (CVD) leads to high morbidity and mortality rate worldwide. Therefore, it is important to determine the risk of CVD across the sociodemographic factors to strategize preventive measures. The current study consisted of 53,122 adults between the ages of 35 and 65 years from The Malaysian Cohort project during recruitment phase from year 2006 to year 2012. Sociodemographic profile and physical activity level were assessed via self-reported questionnaire, whereas relevant CVD-related biomarkers and biophysical variables were measured to determine the Framingham Risk Score (FRS). The main outcome was the 10-year risk of CVD via FRS calculated based on lipid profile and body mass index (BMI) associated formulae. The BMI-based formula yielded a higher estimation of 10-year CVD risk than the lipid profile-based formula in the study for both males (median = 13.2% and 12.7%, respectively) and females (median = 4.3% and 4.2%, respectively). The subgroup with the highest risk for 10-year CVD events (based on both FRS formulae) was the Malay males who have lower education level and low physical activity level. Future strategies for the reduction of CVD risk should focus on screening via BMI-based FRS in this at-risk subpopulation to increase the cost-effectiveness of the prevention initiatives.

## 1. Introduction

Cardiovascular disease (CVD) remains among the most important chronic diseases with a high morbidity and mortality rate worldwide. It is estimated that up to 17.3 million deaths per year globally were due to CVD and these statistics are estimated to reach 23.6 million by the year 2030 [1]. The CVD trend is increasing in developing nations, although it has been decreasing in developed countries [2]. Most Asian countries (except for Japan, South Korea, Singapore, and Thailand) have higher age-adjusted mortality rates from CVD when compared to Western countries. Unlike many of its less developed Asian counterparts, the CVD mortality in Japan has decreased continuously throughout the years, from 40% of all deaths in 1980 to 25% in 2011 [3]. Comparatively, it is quite hard to determine the trend in the same period in Malaysia due to the absence of published reports using the same sampling method. Back in 1980, 22.6% of all registered deaths in Peninsular Malaysia were due to CVD [4], whereas, in 2012, 24.7% of all deaths in Malaysian government hospitals were due to CVD [5]. Although these figures looked optimistic for a developing country, a cross-sectional study conducted among patients undergoing Percutaneous Coronary Intervention in the National Heart Institute, Malaysia, showed that the percentage of premature coronary artery disease cases hovered around 10.6% and 18.8% between the years 2000 and 2012[6].

Although CVD remains the leading killer of both males and females worldwide, there are substantial gender differences in the prevalence and burden of CVD as well as their 10-year CVD risk. It is known that CVD is more predominant among males with higher prevalence than females. Gender differences in CVD mortality largely reflect the gender differences in demographics data. Being female is associated with a longer life expectancy than being male, where females constitute a larger proportion of the elderly population in which the prevalence of CVD is the greatest [7]. Hence, the risk of CVD in females is often underestimated due to the misperception that females are protected against it. The underrecognition of CVD and the differences in their clinical presentation in females have led to a less aggressive treatment among this gender [8]. Therefore, the effort to differentiate the clinical presentation and CVD risk prediction between the genders plays a role in the planning of sex-specific clinical management across the different sociodemographic factors to reduce CVD-related mortality.

The assessment of CVD risk factors has been a key element in the efforts to define a working predictive model for CVD. Absolute risk estimates may serve not only as a basis for decision-making during treatment, but also as a useful approach for risk communication with the affected individuals [9]. The Framingham Heart Study has recognized that CVD predictive model is basically multifactorial, whereby sex, age, systolic blood pressure, total cholesterol, high-density lipoprotein cholesterol, smoking behavior, and diabetes status formed the core variables in the 10-year CVD risk model [10]. Further investigation of the 10-year prediction of CVD in the local setting will help in strategizing the prevention and control of this disease in Malaysia. Hence, the objectives of this study were to determine the difference of 10-year risk estimates of CVD (based on two different Framingham Risk Score (FRS) formulae—based on lipid profile and body mass index (BMI) equations) according to ethnicity, education level, locality, and physical activity across the genders.

## 2. Methods

### 2.1. The Malaysian Cohort (TMC) Study Design and Population

TMC was initiated in 2006 as a nationwide prospective cohort project for the collection of health-related data among approximately 106,527 citizens across Malaysia. The participants comprised multiethnic groups across the different social strata and niches in Malaysia, ranging from 35 to 70 years of age at baseline. The recruitment stage of TMC which started in April 2006 until September 2012 employed multiapproach sampling methods—purposive, cluster, and targeted sampling techniques—to ensure representativeness of the general Malaysian population. To enable a comprehensive examination of the health-related variables among the participants, collection of data came in the form of a guided-interview with a standardized questionnaire, as well as data from relevant biophysical measurements (height, weight, and systolic blood pressure) and blood tests (total cholesterol and high-density lipoprotein (HDL) level). A more thorough description of the TMC study design has been published in another article for convenience [11]. Ethical approval was given by the institutional review and ethics board of the Universiti Kebangsaan Malaysia (Project Code: FF-205-2007) and written informed consent was collected from each participant who joined the project.

### 2.2. Sample Selection

A total of 53,122 participants, age between 35 and 65 years at baseline, were included in this study. Missing data were substantively due to the physical activity variable, resulting from a transition between two versions of physical activity questionnaires during the study (from International Physical Activity Questionnaire to the modified Malay Language Version of the IPAQ-M [12] which was started in January 2010)

### 2.3. Main Outcome

The main outcome of this study was the FRS, which was used to determine the risk estimate of general CVD based on the formula and conversion tables explained by D'Agostino et al. [13]. Their generated risk functions of sex-specific general CVD risk prediction model encompass the risk of coronary heart disease, stroke, intermittent claudication, and congestive heart failure that can be used in the primary care setting. The risk functions were calculated based on age, total cholesterol level, HDL level, systolic blood pressure (based on 2 categories—with hypertension treatment and without hypertension treatment), smoker status, and diabetes status. For this study, two sex-specific CVD risk-related parameters were calculated: (i) 10-year risk estimate of CVD based on FRS using the formula based on lipid profile; (ii) 10-year risk estimate of CVD based on FRS using the formula based on BMI. The score based on BMI-based formula was introduced by D'Agostino et al. for an easier adoption in the field for public health use [13].

All these risk-related parameters comprised continuous data and they were initially log-transformed to address the nonnormality issue during analyses. These log-transformed parameters were then retransformed back as geometric mean estimates of risk (in percentage) for the convenience during the interpretation of the results.

### 2.4. Main Independent Variables

For the sociodemographic factors, the four factors focused on were as follows: (i) age; (ii) ethnicity; (iii) education level; and (iv) locality. Age was defined as the participant's age at baseline during recruitment into the TMC project. This factor was analyzed as a continuous variable in years. Ethnicity was defined based on the self-reported grandpaternal ethnic category. The education level was defined as the highest education level received by the participant at the point of recruitment. The locality of the participant was defined as the predetermined urbanization category of recruitment location, either urban or rural locality.

For the physical activity factor, the level of physical activity was based on the calculation of total Metabolic Equivalent of Task (MET) minutes per week using the IPAQ-M. Validity and reliability of the questionnaires have been described in subcohort study in TMC (12). Physical activity was categorized into high, moderate, and low based on the latest MET categorization provided by the IPAQ Executive Committee [14].

### 2.5. Statistical Methods

Descriptive statistical analysis using mean with standard deviation was done to describe the sample included in the study for normally distributed data. Median and interquartile range (IQR) were used for nonnormally distributed data (due to high skewness or kurtosis values above +2 or below -2). For the inferential statistical analysis, multifactorial analysis of covariance (ANCOVA) via general linear modeling was done [15]. This multivariable statistical analysis was performed to investigate the difference of the 10-year CVD risk estimates based on the chosen factors.

In the multifactorial ANCOVA, we adjusted all the categorical independent variables (ethnicity, locality, education level, and physical activity together with age as the sole continuous independent variable) in a single model. This method yielded an estimate of the adjusted mean or geometric mean for each level of each factor when all these factors (and the accompanying interactions) controlled each other. Effect size was then reported as partial eta squared (*η*^2^) for each independent variable. The effect size was considered to be (i) small if partial *η*^2^ was around 0.01, (ii) medium if partial *η*^2^ was around 0.06, (iii) large if partial *η*^2^ was around 0.14, and very large if partial *η*^2^ was around 0.21 (adapted from Leech et al.) [16].

The significance level was defined as* P *< 0.05 (2-tailed). The related assumptions were tested to ensure the validity of all statistical models [15]. All statistical analyses were carried out using SPSS software version 22 (IBM Corp., Armonk, NY, USA).

## 3. Results

The 53,122 selected individuals from the original 106, 527 TMC participants had the mean age between 48 and 50 years. The BMI-based formula yielded a higher estimation of 10-year CVD risk than the lipid profile-based formula in the study for both males (median (IQR) = 13.2% (14.0%) and 12.7% (13.6%), respectively) and females (median (IQR) = 4.3% (5.9%) and 4.2% (5.6%), respectively). The higher CVD risk among males might be due to the higher prevalence of smoking, high systolic blood pressure, low HDL level, and higher prevalence of diabetics among males as compared to females (Table 1).

As shown in Tables 2 and 3, the 10-year CVD risk was overestimated by the FRS via BMI-based calculation method if compared to the lipid profile-based method, for both genders and other factors in general. Males have a higher 10-year CVD risk, when compared to females, across all the studied factors. The adjusted 10-year CVD risk among males was significantly higher among the Malay ethnic group (geometric mean estimate = 13.4% (95% CI: 13.2-13.6%)), whereas the Chinese (geometric mean estimate = 11.0% (95% CI: 10.7-11.3%)) had the lowest risk based on the lipid profile formula. Similar findings were also found based on the FRS calculated using the BMI-based formula. Males with higher education level have the lowest adjusted CVD risk for both lipid profile-based and BMI-based formulae (geometric mean estimate = 11.7% (95% CI: 11.4-11.9%) and 12.4% (95% CI: 12.1-12.6%), respectively). Males who were staying in an urban locality did not have a higher adjusted CVD risk for both lipid profile-based and BMI-based formulae when compared to a rural locality. Among the males, a small difference was seen for the adjusted CVD risk between those with moderate and high physical activity level, but males with high physical activity level have significantly lower CVD risk based on both the lipid profile-based formulae (geometric mean estimate = 12.2% (95% CI: 12.0-12.4%)), if compared to those who have low physical activity level (Table 2). For the females, the findings for the factors studied were similar to the males, except for the effect of their physical activity level on the adjusted CVD risk. No protective effect was seen among females who have moderate or high physical activity levels (compared to those with low level) in the reduction of their adjusted 10-year CVD risk for both lipid profile-based and BMI-based formulae (Table 3). Besides that, there was a slightly higher significant adjusted CVD risk for urban females (geometric mean estimate = 4.7% (95% CI: 4.6-4.7%)) when compared to rural females (geometric mean estimate = 4.6% (95% CI: 4.5-4.6%)) if BMI-based formula was used.

## 4. Discussion

The analysis of the selected Malaysian Cohort population showed a high 10-year CVD risk among males, Malay ethnic group, those with lower education level, and those with low physical activity level. Urbanity was only shown to be a risk factor in this study if the BMI-based formula was used. The CVD risk factors were more prominent in males as compared to females, whereas the overestimation of 10-year risk CVD could be clearly seen in the FRS calculated based on the BMI formula if compared to the lipid profile formula.

The overestimation of CVD risk via BMI-based FRS in the current study was consistent with the findings by Gray et al. back in 2014, which showed the overestimation of CVD risk for FRS based on BMI-based formula [17]. The BMI-based formula requires the fewest clinical measurements, whereas the lipid profile-based formula requires the measurement of HDL and cholesterol level that are influenced by obesity measured by BMI [18]. Furthermore, obesity is an independent risk factor for fatal coronary events [19]. Hence, the choice of which FRS formula to use depends on the screening setting. For the decision-making of earlier primary and secondary prevention activities in the general community, FRS based on BMI might be the best since the overestimation of CVD risk will lead to a rapid respond of awareness among the targeted individuals. As an aftermath of such prevention activities, successful management of overweight and obesity problems will lead to the reduction of the CVD risk [20].

Overall results clearly showed that males have a higher 10-year CVD risk across the different factors under study (FRS estimate based on lipid profile: 11.0-13.4%; FRS estimate based on BMI: 12.1-13.5%) when compared to females (FRS estimate based on lipid profile: 3.7-5.0%, FRS estimate based on BMI: 4.0-5.1%). This was not surprising as the CVD risk factors among males were consistently worse than females, especially, based on the higher prevalence of smoking status, higher systolic blood pressure, lower HDL level, and higher diabetes comorbidity status. A recent study in 2014 conducted among European diabetics [21] also demonstrated similar findings, with females having a lower 10-year CVD risk compared to males (13.4% and 18.5%, respectively). Conversely, a study among the Jamaican population showed no significant difference of the 10-year CVD risk between the genders [22]. In that study, other factors were postulated to play a more important role as the Jamaican females have a higher prevalence of CVD risk factors compared to the male counterpart. Females with clinically manifested CVD were generally older than the affected males, with a higher expression of CVD risk factors [23]. The age factor is an important differentiating point between males and females in terms of CVD risk because reproductive factors contribute to a lower risk of CVD among females, especially before the menopausal stage [8].

The prevalence of CVD varies according to ethnicity, whereby a recent Community House Project study [24] and a study among the attendees of a primary care clinic [25] in Malaysia showed that the Malays have the highest prevalence of CVD if compared to other ethnic groups, which is consistent with the 10-year CVD risk among the Malays found in the current study. The Malays have more CVD risk factors (e.g., higher prevalence of hypertension, obesity, and hyperlipidemia) if compared to the Indians and the Chinese [26].

The 10-year risk of CVD was observed to be lower among individuals with higher education level for both genders in the current study. If compared to individuals with tertiary education, those with no formal education have higher prevalence in more than three CVD risk factors in Malaysia—including hypercholesterolemia, obesity, and smoking status [26]. This will consequentially lead to a higher CVD risk among those with lower education. In a similar note, an Australian population-based study also found that lower educational attainment which was closely linked to lower socioeconomic status was associated with an increased risk of developing overweight/obesity condition and diabetes, which led to an increased CVD risk [27]. A similar finding was also observed in China [28].

Although the current study failed to consistently demonstrate the effect of urbanity on the risk of CVD across the different models, this factor was still retained in the final model as demonstrated in many past studies in developing countries. For example, there was significant difference of the CVD risk factors between rural and urban populations in Sub-Saharan Africa where CVD was prevalent [29]. In this African study, obesity, high blood pressure, and diabetes were more frequent in urbanized areas, which led to a higher CVD risk. Urban communities might be more exposed to unhealthy lifestyle that contributed to a higher number of CVD risk factors among individuals. A similar observation in a Peruvian population indicates that urbanization is detrimental to cardiovascular health [30]. In India, urban populations with higher socioeconomic status (as reflected by household possessions) were associated with a more adverse CVD risk factors profile [31].

There is evidence in the literature to indicate that physically active individuals have lower rates of CVD [32]. Based on the lipid profile-based and BMI-based FRS for both genders in the current study, individuals with a high level of physical activity status have a significant difference in the 10-year risk of CVD if compared to those with low level of physical activity, but it is not the case for those with moderate activity level. This finding is partially supported by a study in 2013, which showed that a combination of low physical activity and prolonged sitting behavior augmented CVD risk [33]. It should be noted that not all studies demonstrated this cardiovascular protective effect of physical activity. A recent study demonstrated no significant association between physical activity level and the 10-year CVD risk for both genders [34]. Researchers in the future need to take into account all the important biases when assessing the association of physical activity factor and CVD as the results were not always consistent due to various factors that are beyond the control of such studies. However, we can take heart from the fact that most of previous studies have consistently showed that higher physical activity will reduce the modifiable CVD risk factors. It is a fortunate thing seeing that lifestyle modification via increasing physical activity is one of the few cost-effective health improvement measures that remains accessible to everyone, even the poorest [35].

## 5. Conclusions

The current paper has highlighted that the subgroup with the highest risk for 10-year CVD events was the Malay males who have lower education level and low physical activity level. Future strategies for the reduction of CVD risk should focus on active screening via BMI-based FRS in this at-risk subpopulation to increase the cost-effectiveness of the related prevention initiatives. However, there is still a need to include more factors for the refinement of the CVD predictive model to suit the diversity of the Malaysian population. Some relevant confounding factors that were not part of the FRS formulae, such as the waist circumference and ethnicity, might lead to inaccuracy of the prediction of CVD risk. The current paper can be seen as a first step in profiling the Malaysian population in terms of the 10-year risk of CVD based on the predictive model derived from the iconic Framingham Heart Study before the attempt to develop a better predictive model by the end of the follow-up phase of The Malaysian Cohort project.

## Figures and Tables

**Table 1:** Descriptive comparison between the genders for the Framingham Risk Score related variables based on the baseline of The Malaysian Cohort subjects, 2006-2012 (n=53,122).

**Characteristics**	**Male** **(n=22,663)**				**Female** **(n=30,459)**			
	**Mean**	**SD**	%	**95% CI of **%	**Mean**	**SD**	%	**95**%** CI of **%
Age (in years)	49.72	7.85			48.27	7.63		
Systolic blood pressure (mmHg)	128.92	17.00			124.27	19.37		
Total cholesterol (mg/dL)	215.48^a^	53.93^b^			213.67^a^	52.70^b^		
HDL (mg/dL)	46.06^a^	14.81^b^			56.84^a^	20.53^b^		
Total cholesterol: HDL (ratio)	4.80	1.37			3.91	1.17		
BMI (kg/m^2^)	25.53^a^	5.07^b^			25.69^a^	6.45^b^		
Being smoker			30.9	30.3 - 31.5			1.4	1.3 - 1.6
Being diabetic			18.3	17.8 - 18.8			13.2	12.9 - 13.6
10-year risk estimate of CVD based on lipid profile-based formula (%)	12.7^a^	13.6^b^			4.2^a^	5.6^b^		
10-year risk estimate of CVD based on BMI-based formula (%)	13.2^a^	14.0^b^			4.3^a^	5.9^b^		

CI, confidence interval; F, frequency; n, sample size; SD, standard deviation.

^a^Median.

^b^Interquartile range.

**Table 2:** Factors associated with 10-year CVD risk estimate among males based on the baseline of The Malaysian Cohort subjects, 2006-2012 (n=22,663).

**Factors**	**10-year CVD risk estimate based on lipid profile**				**10-year CVD risk estimate based on BMI**			
	**Adjuste** **d** ^**a**^ ** geometric mean estimate of risk **%	**95**%** CI**	**Effect size (partial ** **η** ^2^ **)**	**p-value**	**Adjuste** **d** ^**a**^ ** geometric mean estimate of risk **%	**95**%** CI**	**Effect size (partial ** **η** ^2^ **)**	**p-value**
Ethnicity			0.009	<0.001			0.003	<0.001
Malay	13.4	13.2 - 13.6		Ref.	13.5	13.3 - 13.7		Ref.
(n=8,868)								
Chinese	11.0	10.7 - 11.3		<0.001	12.1	11.8 - 12.4		<0.001
(n=6,094)								
Indian	13.2	12.9 - 13.5		≈1.000	13.4	13.2 - 13.7		≈1.000
(n=4,744)								
Others	12.1	11.8 - 12.3		<0.001	13.2	12.9 - 13.4		0.216
(n=2,957)								
Education Level			0.002	<0.001			0.002	<0.001
No Schooling /	12.9	12.6 - 13.1		Ref.	13.5	13.3 - 13.8		Ref.
Primary School								
(n=4,897)								
Secondary School	12.6	12.4 - 12.8		0.285	13.2	13.0 - 13.4		0.061
(n=11,139)								
University / College	11.7	11.4 - 11.9		<0.001	12.4	12.1 - 12.6		<0.001
(n=6,627)								
Locality			<0.001	0.471			<0.001	0.074
Rural	12.3	12.1 - 12.6		Ref.	12.9	12.7 - 13.1		Ref.
(n=5,015)								
Urban	12.4	12.3 - 12.6		0.471	13.1	13.0 - 13.3		0.074
(n=17,648)								
Physical Activity Level			<0.001	0.010			<0.001	0.088
Low	12.7	12.4 - 12.9		Ref.	13.2	13.0 - 13.4		Ref.
(n=7,425)								
Moderate	12.3	12.1 - 12.5		0.081	12.9	12.7 - 13.2		0.296
(n=7,063)								
High	12.2	12.0 - 12.4		0.010	12.9	12.7 - 13.1		0.105
(n=8,175)								

CI, confidence interval; CVD, cardiovascular disease; n, sample size; Ref, reference group.

^a^Also adjusted for age, each tested factor, and interactions between each tested factor.

**Table 3:** Factors associated with 10-year CVD risk estimate among females based on the baseline of The Malaysian Cohort subjects, 2006-2012 (n=30,459).

**Factors**	**10-year CVD risk estimate based on lipid profile**				**10-year CVD risk estimate based on BMI**			
	**Adjuste** **d** ^**a**^ ** geometric mean estimate of risk **%	**95**%** CI**	**Effect size (partial ** **η** ^2^ **)**	**p-value**	**Adjuste** **d** ^**a**^ ** geometric mean estimate of risk **%	**95**%** CI**	**Effect size (partial ** **η** ^2^ **)**	**p-value**
Ethnicity			0.008	<0.001			0.007	<0.001
Malay	4.8	4.7 - 4.9		Ref.	4.9	4.8 - 4.9		Ref.
(n=10,873)								
Chinese	3.7	3.6 - 3.8		<0.001	4.0	3.9 - 4.1		<0.001
(n=9,818)								
Indian	4.6	4.5 - 4.7		0.030	4.7	4.6 - 4.8		0.035
(n=5,719)								
Others	4.7	4.6 - 4.8		≈1.000	5.0	4.9 - 5.1		0.187
(n=4,049)								
Education Level			0.006	<0.001			0.006	<0.001
No Schooling /	5.0	4.9 - 5.0		Ref.	5.1	5.0 - 5.2		Ref.
Primary School								
(n=8,473)								
Secondary School	4.4	4.3 - 4.5		<0.001	4.6	4.5 - 4.6		<0.001
(n=14,875)								
University / College	4.0	3.9 - 4.1		<0.001	4.3	4.1 - 4.3		<0.001
(n=7,111)								
Locality			<0.001	0.052			<0.001	0.036
Rural	4.4	4.3 - 4.5		Ref.	4.6	4.5 - 4.6		Ref.
(n=6,538)								
Urban	4.5	4.4 - 4.5		0.052	4.7	4.6 - 4.7		0.036
(n=23,921)								
Physical Activity Level			<0.001	0.064			<0.001	0.179
Low	4.5	4.4 - 4.6		Ref.	4.7	4.6 - 4.8		Ref.
(n=7,775)								
Moderate	4.4	4.3 - 4.5		0.087	4.6	4.5 - 4.6		0.257
(n=13,112)								
High	4.4	4.3 - 4.5		0.122	4.6	4.5 - 4.7		0.312
(n=9,572)								

CI, confidence interval; CVD, cardiovascular disease; n, sample size; Ref, reference group.

^a^Also adjusted for age, each tested factor, and interactions between each tested factor.

## Data Availability

Information on The Malaysian Cohort is available at www.ukm.my/mycohort/ms/. Requests for other data or information can be made to the corresponding author via e-mail. The authors welcome national and international collaborations and proposals can be forwarded to rahmanj@ppukm. ukm.edu.my, and they will then be discussed at the steering committee.
